# High prevalence of diabetes and intermediate hyperglycemia – The Brazilian Longitudinal Study of Adult Health (ELSA-Brasil)

**DOI:** 10.1186/1758-5996-6-123

**Published:** 2014-11-18

**Authors:** Maria Inês Schmidt, Juliana F Hoffmann, Maria de Fátima Sander Diniz, Paulo A Lotufo, Rosane Härter Griep, Isabela M Bensenor, José G Mill, Sandhi Maria Barreto, Estela M L Aquino, Bruce B Duncan

**Affiliations:** Postgraduate Studies Program in Epidemiology, School of Medicine, Federal University of Rio Grande do Sul, Av. Ramiro Barcelos, 2600/414, 90035-003 Porto Alegre, RS Brazil; Faculdade de Medicina, Federal University of Minas Gerais, Belo Horizonte, Brazil; Center for Clinical and Epidemiologic Research, University of São Paulo, São Paulo, Brazil; Laboratory of Health and Environment Education, Fundação Oswaldo Cruz, Rio de Janeiro, Brazil; Department of Physiological Sciences, Federal University of Espírito Santo, Vitória, Brazil; Instituto Saúde Coletiva, Federal University of Bahia, Salvador, Brazil

**Keywords:** Diabetes mellitus, Prevalence, Brazil, Prediabetic state, Hyperglycemia

## Abstract

**Background:**

The global burden of diabetes mellitus and other chronic diseases is high, and 80% of those with diabetes now live in low and middle income countries. Yet, little information is available regarding prevalence of diabetes and intermediate hyperglycemia in these countries, especially when a full range of diagnostic tests is employed. The purpose of this study is to provide a full accounting of these prevalences in a large, free-living Brazilian population.

**Methods:**

We report baseline data (2008-2010) from the Brazilian Longitudinal Study of Adult Health (ELSA-Brasil), a cohort study of 15,105 civil servants aged 35-74 years. Diabetes mellitus was ascertained by self-report of diagnosis, medication use, fasting glucose, an oral glucose tolerance test, and/or glycated hemoglobin. Cut-offs for diabetes and intermediate hyperglycemia followed the recommendations of the World Health Organization and the American Diabetes Association. Adjusted prevalences were estimated through logistic regression.

**Findings:**

With this full accounting, 19.7% (19.0%-20.3%) had diabetes mellitus, 50.4% being previously undiagnosed. Frequencies of intermediate hyperglycemia according to various criteria ranged from 16.1% to 52.6%. Diabetes or intermediate hyperglycemia was present in 79.1% of participants when using the most comprehensive definitions. The burden was greatest in the elderly, the obese, non-whites, and those with less formal education (p < 0.001).

**Interpretation:**

That four of every five free-living individuals aged 35-74 years working in selected public institutions in six Brazilian state capitals presented either diabetes or intermediate hyperglycemia highlights the advanced stage of the obesity – diabetes epidemic in urban Brazil and indicates the need for urgent action.

## Introduction

The United Nations has declared that the global burden of chronic non-communicable diseases (NCDs) constitutes one of the major challenges for development in the 21^st^ century, particularly for developing countries. Diabetes mellitus (diabetes) along with cancer, cardiovascular and chronic respiratory diseases represent about 80% of the NCD mortality [1]. Additionally, diabetes is one of the 10 main causes of global disease burden when evaluated by years of life lived with disability [2].

The prevalence of diabetes has risen dramatically in the last decades. The International Diabetes Federation (IDF) estimated that 382 million people in 2013 had diabetes, 175 million (46%) being undiagnosed [3]. Based on the IDF estimates, 80% of the cases of diabetes globally live in low and middle income countries. In absolute numbers, Brazil was ranked 4^th^ among all nations. Yet, prevalence of diabetes in Brazil has been largely based on self-reported cases, given the difficulties in conducting large surveys with laboratory determinations.

The only Brazilian study which included blood glucose measurement and was not based in just one local setting, conducted between 1986 and 1988, found a prevalence of 7.6% in adults 30-69 years of age of various capitals of different geographic regions [4]. This prevalence certainly underestimates current rates given the epidemiologic and nutritional transitions that have occurred in Brazil over the last decades [5]. Estimates for impaired glucose tolerance (IGT) [3] and other categories of risk based on fasting glucose (impaired fasting glucose, IFG) or glycated hemoglobin (elevated HbA_1C_) [6], frequently referred to as intermediate hyperglycemia or prediabetes, are also either outdated or restricted to local settings.

Thus, to gain insight in terms of the current burden of diabetes and intermediate hyperglycemia with a full accounting of diagnostic laboratory exams (fasting glucose, a 75 g oral glucose tolerance test and a measurement of HbA_1C_), the objective of this study is to describe their prevalence, overall and according to sociodemographic and nutritional variables, in the baseline examination of a large cohort of Brazilian adults conducted in six capitals of Brazil, thus allowing for a broad representation of major relevant population groups.

## Methods

The Brazilian Longitudinal Study of Adult Health (ELSA-Brasil) is a prospective cohort study designed to investigate diabetes, cardiovascular and other related chronic diseases, as previously described [7, 8]. The ethics committee of all institutions approved the research protocol, and all participants gave written consent.

Active or retired civil servants (35-74 years) of universities or research institutions from six state capitals of three different regions of Brazil were invited to participate, totalizing, in 2008, 52,137 potential volunteers. Men, the elderly, and those whose activities did not require a higher education were especially encouraged to participate. A total of 16,435 expressed interest, 15,821 being pre-enrolled. Between 2008 and 2010, 15,105 subjects completed the baseline examination. After excluding three participants who lacked laboratory values, our final sample for analyses comprises 15,102 participants.

A previously validated, comprehensive set of questionnaires, clinical measurements and laboratory tests was carried out. Sociodemographic factors and past medical history were ascertained by questionnaire. Participants were requested to bring their medications or prescriptions to the clinic. Weight and height were measured using standard equipment and techniques. A 12-hour fasting blood sample was drawn in the morning soon after arrival at the research clinic, following standardized procedures for samples collection and processing [9]. A standardized 75 g oral glucose tolerance test (OGTT) was performed in all participants without known diabetes utilizing an anhydrous glucose solution [10]. Plasma glucose was measured by the hexokinase method (ADVIA Chemistry; Siemens, Deerfield, Illinois). HbA_1C_ was measured by high-pressure liquid chromatography (Bio-Rad Laboratories, Hercules, California), using a method certified by the National Glycohemoglobin Standardization Program.

Diabetes status was defined in a comprehensive fashion, as follows. A previously diagnosed diabetes was classified when answering, “yes” to either “Have you been previously told by a physician that you had/have diabetes (sugar in the blood)?” or “Have you used medication for diabetes in the past 2 weeks?” Previously undiagnosed diabetes was classified based on laboratory values when reaching the threshold for fasting plasma glucose (FPG; ≥7.0 mmol/L), 2-hour plasma glucose during the OGTT (2 h PG ≥11.1 mmol/L), or HbA_1C_ (≥6.5%; ≥47.5 mmol/mol) [11–13].

More restrictive secondary definitions were also calculated: first, by excluding those classified only on the basis of their HbA_1C_ value, since this test has only recently been recommended for diagnostic purposes; second, by only considering FPG, as this is the approach most frequently used in clinical practice; and finally by also requiring that the participant’s report of a previous diagnosis be coupled with either medication use or laboratory confirmation.

Intermediate hyperglycemic states, among those not meeting the comprehensive diabetes definition, used the following cut-offs: impaired glucose tolerance (IGT), a 2 h PG ≥7.8 mmol/L; impaired fasting glucose (IFG; by the World Health Organization – WHO – criteria), a FPG ≥6.1 mmol/L [13], or alternatively a FPG ≥5.6 mmol/L, according to the American Diabetes Association [11]; and elevated HbA_1C,_ a value ≥5.7% (≥38.8 mmol) [11] or, alternatively ≥6.0% (≥42.1 mmol) [14].

Race was defined by the participant’s self-declared skin color/race. Educational level was ascertained in years and then classified in levels. Body mass index (BMI) was defined as weight (kg)/height (m^2^) and classified according to the standard definition.

We estimated crude and adjusted prevalences and their 95% confidence intervals through logistic regression using the procedure PROC RLOGIST and the predmarg statement in SUDAAN. Smoothing the lines of prevalence across age was performed using a spline routine (proc gploc; interpol = sm30, SAS). All analyses were done using Statistical Analysis System (SAS 9.3 for Windows software; SAS Institute, Cary, NC) and SUDAAN version 11.0. A p-value of less than 0.05 was considered statistically significant.

## Results

At baseline, mean age was 52.1 years and mean BMI 27.0 kg/m^2^. As seen in Table 1, we enrolled large numbers of men and women, of the four age categories and of participants declaring as being one of the main three race/color categories of the Brazilian population. Of note, 52.6% had a university degree and 63.1% were either overweight or obese.

**Table 1:** **Selected socio-demographic and nutritional characteristics of study participants during the baseline of the Brazilian Longitudinal Study of Adult Health (ELSA-Brasil), 2008-2010**

	n	%	95% CI
**Gender (n = 15102)**			
Men	6885	45.6	44.8-46.4
Women	8217	54.4	53.6-55.2
**Age (n = 15102)**			
35-44	3340	22.1	21.5-22.8
45-54	5937	39.3	38.5-40.1
55-64	4233	28.0	27.3-28.8
65-74	1592	10.5	10.1-11.0
**Self-identified skin color/race category (n = 14918)**			
White	7789	52.2	51.4-53.0
Black	2397	16.1	15.5-16.7
Brown (“pardo”)	4201	28.2	27.4-28.9
Asian	374	2.5	2.3-2.8
Indigenous	157	1.1	0.9-1.2
**Educational level (n = 15102)**			
Incomplete elementary school	894	5.9	5.5-6.3
Incomplete secondary school	1028	6.8	6.4-7.2
Secondary school	5232	34.6	33.9-35.4
University degree	7948	52.6	51.8-53.4
**Body mass index categories (BMI) (n = 15096)**			
Underweight (<18.5 kg/m^2^)	143	1.0	0.8-1.1
Normal ( 18.5-24.9 kg/m^2^)	5422	35.9	35.2-36.7
Overweight (25-29.9 kg/m^2^)	6073	40.2	39.5-41.0
Obese (≥30 kg/m^2^)	3458	22.9	22.2-23.6

Small differences in total frequency (n) of characteristics are due to missing values.

As shown in Table 2, when utilizing all of the diagnostic information available (definition 1a), the prevalence of diabetes was 19.7% (19.0%-20.3%). By restricting the classification of self-reported diabetes (definition 2a) to cases reporting previous diagnosis combined with either medication use or confirmation by laboratory testing in ELSA, prevalence decreased to 18.4% (17.8%-19.0%). The exclusion of HbA_1C_ from the diagnostic criteria decreased total prevalence by 1.9% for each of the above definitions, to 17.8% (17.2%-18.4%) and to 16.5% (15.9%-17.1%), respectively. When additionally excluding the 2 h post-load plasma glucose, prevalence decreased to 15.1% (14.5%-15.7%) and 13.6% (13.1%-14.2%), respectively. The percentage of previously diagnosed diabetes was generally similar to that of previously undiagnosed diabetes, except when the latter was defined solely by fasting plasma glucose.

**Table 2:** **Prevalence of diabetes, baseline of the Brazilian Longitudinal Study of Adult Health (ELSA-Brasil), n = 15102, 2008-2010**

Diabetes definitions	Total cases			Cases previously diagnosed			Cases previously undiagnosed			Percent undiagnosed*	
	n	%	95% CI	n	%	95% CI	n	%	95% CI	%	95% CI
1. Previously diagnosed (self-report or medication use) *or* undiagnosed reaching:											
a. diabetes cut-off for HbA_1C_, FPG or 2hPG	2970	19.7	19.0 – 20.3	1473	9.8	9.3 – 10.2	1497	9.9	9.4 – 10.4	50.4	48.6-52.2
b. diabetes cut-off for FPG or 2hPG	2688	17.8	17.2 – 18.4	1473	9.8	9.3 – 10.2	1215	8.0	7.6 – 8.5	45.2	43.3-47.1
c. diabetes cut-off for FPG	2280	15.1	14.5 – 15.7	1473	9.8	9.3 – 10.2	807	5.3	5.0 – 5.7	35.4	33.4-37.4
2. Previously diagnosed (restricted definition**) *or* undiagnosed reaching:											
a. diabetes cut-off for HbA_1C_, FPG or 2hPG	2778	18.4	17.8 – 19.0	1202	8.0	7.5 – 8.4	1576	10.4	10.0 – 10.9	56.7	54.9-58.6
b. diabetes cut-off for FPG or 2hPG	2486	16.5	15.9 – 17.1	1202	8.0	7.5 – 8.4	1284	8.5	8.1 – 9.0	51.7	49.7-53.6
c. diabetes cut-off for FPG	2055	13.6	13.1 – 14.2	1202	8.0	7.5 – 8.4	853	5.7	5.3 – 6.0	41.5	39.4-43.6

HbA_1C_ = glycated hemoglobin; FPG = fasting plasma glucose; 2hPG = plasma glucose 2 h after a glucose load.

*Cases previously undiagnosed / Total cases.

**Requiring that a previous diagnosis of diabetes be accompanied by medication use or be verified by a laboratory result.

Diabetes cut offs: FPG (≥7.0 mmol/L); 2-hour PG (≥11.1 mmol/L); or HbA1C (≥6.5%; ≥47.5 mmol/mol).

Frequencies of previously diagnosed and undiagnosed diabetes increased importantly with age among men and women (Table 3). The proportion of those previously undiagnosed (right columns) decreased with increasing age, from 58.3% to 40.6% for the age strata of 35-44 years to 65-74 years.

**Table 3:** Distribution of diabetes cases* according to categories of sex and age, baseline of the Brazilian Longitudinal Study of Adult Health (ELSA-Brasil), n = 15102, 2008-2010

	Previously diagnosed					Previously undiagnosed					Percent undiagnosed		
	Men		Women		Total	Men		Women		Total	Men	Women	Total
Age Strata	n	%	n	%	%	n	%	n	%	%	%	%	%
35-44	60	3.8	39	2.2	3.0	90	5.8	51	2.9	4.2	60.4	56.9	58.3
45-54	236	8.8	202	6.2	7.4	316	11.8	251	7.7	9.6	57.3	55.4	56.5
55-64	315	17.0	289	12.2	14.3	291	15.7	270	11.4	13.3	48.0	48.3	48.2
65-74	169	21.5	163	20.3	20.9	126	16.0	102	12.7	14.3	42.7	38.5	40.6
**Total**	780	11.3	693	8.4	9.8	823	12.0	674	8.2	9.9	51.5	49.4	50.4

*considering a self-reported diagnosis, use of medication, or a full accounting of glucose abnormalities (Definition 1a in Table 1).

As seen in Table 4, the crude prevalence of total and previously diagnosed diabetes was higher among men and increased markedly with age and with increasing BMI. Whites had a lower prevalence of diabetes than other groups. Highest rates were seen in Asian, black and indigenous Brazilians. A smooth gradient was seen in terms of educational attainment, with participants with lesser education having a notably greater prevalence. Adjustment for the above-mentioned variables resulted in the following comparisons: Among the obese the adjusted prevalence was 2.7 times that among the lean; those over 65 had 4.4 times the adjusted prevalence of those under 45; men had a 42.6% greater prevalence than women; blacks a 37.9% and Asians (primarily of Japanese origin) a 61.0% greater adjusted prevalence than whites; and those not completing elementary school a 64.6% greater adjusted prevalence than those with a university education. Prevalence among “browns”, a group with ethnically mixed – Caucasian, African and to a lesser extent indigenous – ancestry, was intermediate between that seen among whites and blacks. Although a generally similar pattern was seen when considering only those previously diagnosed diabetes, differences were accentuated across age and BMI categories; Asian, rather than indigenous Brazilians had the highest adjusted frequency among race/color groups.

**Table 4:** Prevalence of total and previously diagnosed diabetes* according to selected socio-demographic and nutritional characteristics, baseline of the Brazilian Longitudinal Study of Adult Health (ELSA-Brasil), n = 14912, 2008-2010

	Total diabetes						Previously diagnosed diabetes					
	Unadjusted			Adjusted**			Unadjusted			Adjusted**		
Population correlate	n	%	95% CI	%	95% CI	P value	n	%	95% CI	%	95% CI	P value
**Sex**						<0.0001						<0.0001
Men	1572	23.2	22.2-24.2	23.4	22.5-24.4		762	11.2	10.5-12.0	11.4	10.7-12.2	
Women	1350	16.6	15.8-17.4	16.4	15.6-17.2		683	8.4	7.8-9.0	8.3	7.7-8.9	
**Age (years)**						<0.0001						<0.0001
35-44	240	7.2	6.4-8.1	7.9	7.0-8.9		99	3.0	2.4-3.6	3.2	2.7-3.9	
45-54	993	16.9	16.0-17.9	16.5	15.6-17.4		432	7.4	6.7-8.0	7.1	6.5-7.8	
55-64	1142	27.4	26.1-28.8	27.0	25.7-28.3		592	14.2	13.2-15.3	14.1	13.0-15.1	
65-74	547	35.0	32.7-37.4	34.6	32.3-37.0		322	20.6	18.6-22.6	20.8	18.8-22.9	
**Body mass index (BMI)**						<0.0001						<0.0001
<25 kg/m^2^	592	10.8	9.9-11.6	11.7	10.9-12.6		268	4.9	4.3-5.4	5.3	4.8-6.0	
25-29.9 kg/m^2^	1187	19.8	18.8-20.8	18.9	18.0-19.9		603	10.1	9.3-10.8	9.6	8.9-10.3	
≥30 kg/m^2^	1143	33.5	31.9-35.1	32.1	30.6-33.6		574	16.8	15.6-18.1	16.0	14.8-17.2	
**Race/Color**						<0.0001						<0.0001
White	1291	16.6	15.8-17.4	17.7	16.8-18.6		634	8.1	7.5-8.8	8.6	7.9-9.3	
Brown	836	19.9	18.7-21.1	19.3	18.1-20.5		406	9.7	8.8-10.6	9.6	8.7-10.5	
Black	655	27.4	25.6-29.1	24.4	22.7-26.1		329	13.7	12.4-15.1	12.3	11.0-13.6	
Indigenous	45	28.7	21.5-35.8	20.9	15.9-27.0		22	14.0	8.5-19.5	9.8	6.5-14.6	
Asian	95	25.4	21.0-29.8	28.5	24.3-33.1		54	14.4	10.9-18.0	15.7	12.4-19.7	
**Educational attainment**						<0.0001						<0.0001
Incomplete elementary	331	37.7	34.5-40.9	26.5	24.0-29.2		177	20.1	17.5-22.8	13.2	11.4-15.3	
Incomplete secondary	307	30.1	27.3-32.9	23.4	21.2-25.8		154	15.1	12.9-17.3	11.4	9.8-13.2	
Secondary school	1123	21.7	20.5-22.8	22.1	21.0-23.3		555	10.7	9.9-11.6	11.2	10.3-12.1	
University degree	1161	14.8	14.0-15.6	16.1	15.3-17.0		559	7.1	6.6-7.7	7.7	7.1-8.4	

*considering self-reported diagnosis, use of medication, and a full accounting of glucose abnormalities (Definition 1a in Table 1).

**Adjusted for other variables in table and study center.

As shown in Table 5, prevalence of intermediate hyperglycemia varied considerably according to the definition used. The prevalence of IGT (20.3%) was similar to that of IFG based on the WHO criteria (19.1%). However, the prevalence of IFG based on the ADA criteria was much larger (52.6%). Regardless of the criteria used, these intermediate states of hyperglycemia were higher in men. When classified by HbA_1C_ values, intermediate hyperglycemia was present in 16.1% (ADA definition) and in 7% (using the previous Expert Committee recommendation), and was more common among women.

**Table 5:** Prevalence of intermediate states of hyperglycemia,* baseline of the Brazilian Longitudinal Study of Adult Health (ELSA-Brasil), n = 15102, 2008-2010

	Total			Men (n = 6885)			Women (n = 8217)		
	n	%	95% CI	n	%	95% CI	n	%	95% CI
**IGT**	3064	20.3	19.7-20.9	1455	21.1	20.2-22.1	1609	19.6	18.7-20.4
**IFG (WHO)**	2886	19.1	18.5-19.7	1680	24.4	23.4-25.4	1206	14.7	13.9-15.4
**IFG (ADA)**	7946	52.6	51.8-53.4	4050	58.8	57.7-60.0	3896	47.4	46.3-48.5
**Intermediate HbA**_**1C**_ **(ADA**_**)**_ **	2431	16.1	15.5-16.7	1011	14.7	13.9-15.5	1420	17.3	16.5-18.1
**Intermediate HbA**_**1C**_ ≥6.0%***	1049	7.0	6.5-7.4	433	6.3	5.7-6.9	616	7.5	6.9-8.1
**IFG (WHO) or IGT**	4684	31.0	30.3-31.8	2406	35.0	33.8-36.1	2278	27.7	26.8-28.7
**IFG (ADA) or IGT**	8472	56.1	55.3-56.9	4217	61.3	60.1-62.4	4255	51.8	50.7-52.9
**IFG (WHO) or Intermediate HbA**_**1C**_ **(ADA)**	4514	29.9	29.2-30.6	2266	32.9	31.8-34.0	2248	27.4	26.4-28.3
**IFG (ADA) or Intermediate HbA**_**1C**_ **(ADA)**	8532	56.5	55.7-57.3	4210	61.2	60.0-62.3	4322	52.6	51.5-53.7
**IGT, IFG (WHO) or Intermediate HbA**_**1C**_ **(ADA)**	5929	39.3	38.5-40.0	2852	41.4	40.3-42.6	3077	37.5	36.4-38.5
**IGT, IFG (ADA) or Intermediate HbA**_**1C**_ **(ADA)**	8971	59.4	58.6-60.2	4356	63.3	62.1-64.4	4615	56.2	55.1-57.2

*After excluding diabetes defined by a self report, medication use or elevated HbA_1C_, fasting plasma glucose or plasma glucose 2 h after a glucose load.

**HbA_1C_ ≥5.7% (≥38.8 mmol).

***HbA_1C_ ≥6.0% (≥42.1 mmol).

IGT = impaired glucose tolerance; IFG = impaired fasting glucose; ADA = American Diabetes Association; WHO = World Health Organization; HbA_1C_ = glycated hemoglobin.

Considering that tests are frequently done together in the clinical setting, we also analyzed test results jointly. The frequency of intermediate hyperglycemia reached very high values, varying from 29.9% to 59.4% depending on the definition used (Table 5).

Table 6 shows that in both men and women, the frequency of IGT increased by age categories. A similar pattern was observed with IFG defined by the WHO criteria, with the exception of the oldest men. IFG (ADA) decreased by age among men, and did not change much among women over 45 years. Elevated HbA1_C_ increased slightly with age among women, but showed no clear age trend among men. Ethnic differences in the prevalence of intermediate hyperglycemia categorized according to the various definitions were generally small, the exception being for the category defined by HbA_1C_, for which blacks had a considerably greater prevalence.

**Figure 1:**
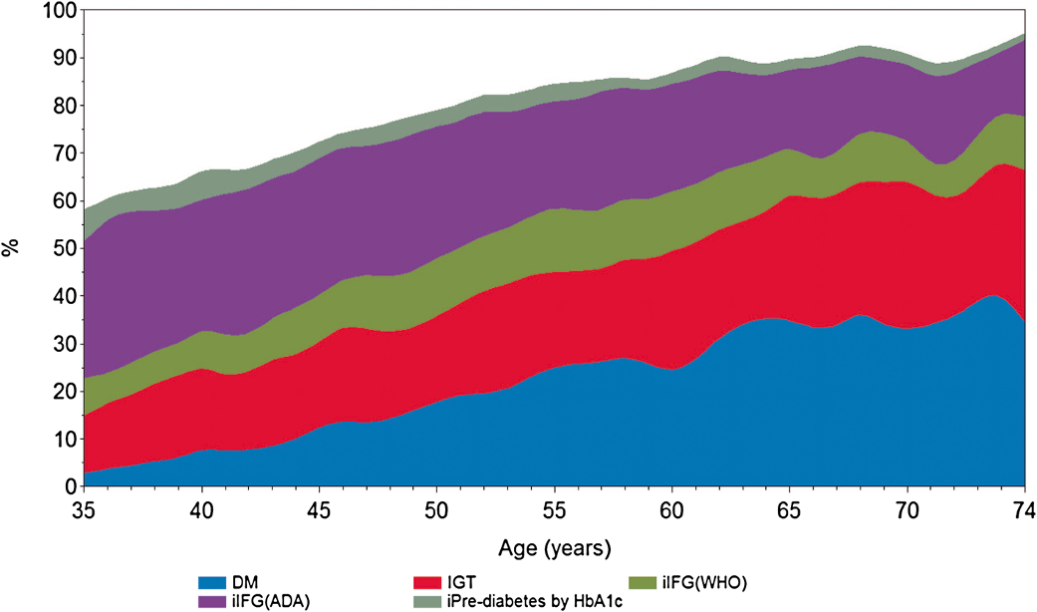
**Percent of the total sample presenting a glucose abnormality by category and age.** Brazilian Longitudinal Study of Adult Health (ELSA-Brasil), N = 15102, 2008-2010. Smoothing performed using a spline routine (Statistical Analysis System). DM = diabetes mellitus; IGT = impaired glucose tolerance; iIFG (WHO) = isolated impaired fasting glucose; iIFG(ADA) represents the additional cases of IFG when ascertained by the ADA criteria; iPre-diabetes by HbA1c represents the additional cases of intermediate hyperglycemia when diagnosed by ADA criteria; WHO = World Health Organization; ADA = American Diabetes Association. See Methods for diagnostic criteria.

Considering diabetes and intermediate hyperglycemia together, more than half of participants presented an abnormality, regardless of the criteria used to define IFG. In fact, when IFG (ADA) was considered, this combined frequency reached 79.1%, leaving only 20.9% of participants classified as having normoglycemia. Figure 1 illustrates the fully accounted burden of diabetes and intermediate hyperglycemia graphically, showing the cumulative percentage of participants presenting these conditions by age. Of note, over 80% of participants aged 50 or greater were affected, and this fraction exceeded 90% in those above age 65.

**Table 6:** Distribution of intermediate hyperglycemia categories* according to sex, race and age, baseline of the Brazilian Longitudinal Study of Adult Health (ELSA-Brasil), n = 15102, 2008-2010

	Impaired glucose tolerance					Impaired fasting glucose (WHO)					Impaired fasting glucose (ADA)					Intermediate HbA _1C_ **				
	Men		Women		Total	Men		Women		Total	Men		Women		Total	Men		Women		Total
	n	%	n	%	%	n	%	n	%	%	n	%	n	%	%	n	%	n	%	%
**Total**	1455	21.1	1609	19.6	20.3	1680	24.4	1206	14.7	19.1	4050	58.8	3896	47.4	52.6	1011	14.7	1420	17.3	16.1
**Age**																				
35-44	263	16.9	286	16.1	16.4	286	18.3	132	7.4	12.5	976	62.5	675	37.9	49.4	240	15.4	244	13.7	14.5
45-54	556	20.7	606	18.6	19.6	681	25.4	471	14.4	19.4	1635	61.0	1605	49.3	54.6	422	15.7	573	17.6	16.8
55-64	428	23.1	477	20.1	21.4	512	27.6	444	18.7	22.6	1028	55.4	1222	51.4	53.2	237	12.8	446	18.8	16.1
65-74	208	26.4	240	29.9	28.1	201	25.5	159	19.8	22.6	411	52.2	394	49.0	50.6	112	14.2	157	19.5	16.9
**Race/Color (n = 14918)**																				
White	804	22.3	828	19.8	21.0	895	24.9	597	14.2	19.2	2226	61.9	2032	48.5	54.7	498	13.8	639	15.3	14.6
Brown	405	20.0	440	20.2	20.1	478	23.6	338	15.5	19.4	1155	57.0	1060	48.7	52.7	292	14.4	390	17.9	16.2
Black	179	19.0	250	17.2	17.9	215	22.9	209	14.4	17.7	489	52.0	617	42.4	46.1	181	19.2	331	22.7	21.4
Indigenous	19	20.7	10	15.4	18.5	23	25.0	14	21.5	23.6	52	56.5	31	47.7	52.9	10	10.9	11	16.9	13.4
Asian	29	22.7	66	26.8	25.4	40	31.3	32	13.0	19.3	69	53.9	119	48.4	50.3	15	11.7	35	14.2	13.4

*After excluding diabetes defined by self-report, medication use or HbA_1C_, FPG or 2hPG.

**HbA_1C_ ≥5.7% (≥38.8 mmol).

## Discussion

The prevalence of diabetes in Brazil assessed by self-reported has increased over the last two decades, in part due to greater access to diagnostic testing [5]. In the late 1980s, the prevalence was 4.1% in individuals 30-69 years residing in various metropolitan areas [4]. In 2006, a telephone survey based on wider age range (18 years or older), found a prevalence of 5.3% among adults residing in Brazilian capital cities [15]. Conducted annually ever since with similar probabilistic samples, this telephone survey found a prevalence of 6.8% by 2012 [16], a 28% jump in just seven years. Of note, when using comparable diabetes definitions (self-reported diagnosis, without consideration of medication use) and age ranges, the prevalence of known diabetes (8.8%) in our cohort, which is based in six of the 22 cities covered by the annual telephone survey, was quite comparable to that observed (9.8%) in the 2010 telephone survey [8].

That intermediate degrees of hyperglycemia were extremely common suggests that the prevalence of diabetes will increase for several years to come. For every one adult with diabetes in our sample, approximately one had IGT (20.3%) and 1.5 IGT or IFG (≥6.1 mol/L; 31.0%). If the lower ADA IFG cut point (≥5.6 mol/L) were used, almost 3 adults (56.1%) would be classified as having either IGT or IFG for every one with diabetes. Data from a recent meta-analysis suggest that over a 10 year period, approximately 1 in 3 of those with intermediate hyperglycemia by IFG (ADA) or HbA_1C_ criteria, almost half of those with intermediate hyperglycemia by IFG (WHO) or with IGT, and 70% of those with IFG and IGT will convert to frank diabetes [6]. The relevance of expanding the WHO definition of IFG to that of the ADA by including fasting glucose in the 100-109 mg/dL range is unclear, and is a major issue, as it labels a very large fraction of the population as being at risk.

Additionally, the disparities in the prevalence of intermediate hyperglycemia when assessed by these different measures and also when assessed by a specific measure in varying age and sex groups suggest that these measures result, to a certain extent, from different processes. Of note in this regard, elevated HbA1c was more common among blacks, which is consistent with previous studies [17].

The time trend indicated by these Brazilian studies is consistent with findings from around the world which show dramatic increases in diabetes prevalence over the past few decades [3, 18]. When a similar age group is used for comparison, our rates of diabetes are higher than those recently reported for China and for Panama [19, 20]. The high prevalences we found for intermediate hyperglycemia are also consistent with those previously reported globally [3, 18, 19, 21]. The much higher prevalence we found for IFG based on the ADA criteria in comparison with those for other intermediate hyperglycemic states has been previously described [22].

The burden of diabetes we observed in specific sub-groups is remarkable. A total of 27.5% of those between 55 and 64 years of age had diabetes, this percentage rising to 35.2% for those aged 65 to 74. Of note, this latter percentage of 35.2% reflects an individual’s probability of developing diabetes across the lifespan, a probability which is not readily perceived when risk is expressed as mean diabetes prevalence among adults ≥18 years of age, especially when this is based on self-report (e.g. 7.4%). Similarly, 33.7% of all obese participants had diabetes. More than 25% of all blacks, those of Asian descent and indigenous Brazilians, and more than 30% of all those with less than a high school education had diabetes. In fact, with full accounting and using the ADA definition of IFG, only 12.8% of obese participants, and only 10.9% of participants greater than 65 years of age, could be classified as having normoglycemia.

A full understanding of the origins of the global epidemic of diabetes and intermediate hyperglycemia has yet to be assembled. Factors possibly related to it, for example, high sugar consumption, have been little investigated in Brazil. However, regardless of the multitude of possible causes involved, there is no doubt that the obesity epidemic has played a major role. Since the obesity burden in Brazil, as in many settings, is shifting toward the poor [23], this may explain, in part, the social gradient here described for diabetes prevalence. Of note, the high prevalence we found among Asian descendents is consistent with previous studies [24].

Meriting discussion also is the proportion of previously undiagnosed diabetes we found, which was similar to that seen in a Brazilian population survey based on results of an OGTT (46%), conducted in the late 1980s [4]. We expected a lower proportion of undiagnosed diabetes in response to the greater access to health services in recent decades. At least two reasons may explain our findings. First, when our undiagnosed cases were ascertained with all possible diagnostic tests, their contribution to overall prevalence was higher than when ascertained only with fasting glucose, which is still the most common clinical practice. In fact, when only fasting plasma glucose was used to define undiagnosed diabetes, the proportion was lower, only 35.4%. Second, the diagnosis of asymptomatic cases of diabetes in clinical practice requires confirmation on a different day, while our classification of undiagnosed diabetes (as in most epidemiologic surveys) was based on only one examination. Based on published estimates [25], this, by itself, could reduce the prevalence of undiagnosed diabetes by 25%. Nevertheless, it is clear that the absolute prevalences of both diagnosed and undiagnosed diabetes are in rapid ascension globally [3], and that most previously undetected cases in our study are likely to represent real cases in the community.

Our study has important strengths, including the size and diversity of the sample studied and the standardized data collection. The capacity to fully account for hyperglycemia and the various means of ascertainment of diabetes and intermediate hyperglycemia permit a comprehensive description of the disease burden across relevant socioeconomic groups, which is rarely accomplished in large surveys, particularly those from low and middle income countries.

Limitations to our study also merit discussion. First, our sample, consisting of university and research institute employees with stable employment and a high educational achievement, is not representative of the entire Brazilian population. However, as already mentioned, in terms of self-reported diabetes prevalence, our findings differ little from those found in a representative sample of similarly aged adults from Brazil’s capital cities [8]. Moreover, since diabetes prevalence is inversely related to educational attainment, if a bias is present in our sample through under-representation of those with less formal education, it is, if anything, likely to be a conservative one, leading to an underestimation of the prevalence of hyperglycemic conditions. Further, since we based our sample on individuals living in metropolitan areas, our results are most readily generalized to urban Brazil, which, according to the 2010 census, is the home of 84% of the Brazilian population [26].

Second, our ascertainment of previously known diabetes may suffer from the increased diabetes and intermediate hyperglycemia screening in recent years, since participants may tell us, erroneously, that they have diabetes when they have lesser than diabetes hyperglycemia. Further, a positive response to the question about diabetes medication use may also inadvertently ascertain diabetes when in fact the medication was being used to prevent, rather than to treat diabetes. However, though they would upwardly bias our prevalences, the effect of these problems is likely to be small, as shown by the minimal differences in prevalence seen comparing definitions 1 and 2 (Table 2).

Within these limitations, we were able to provide an accurate and full ascertainment of diabetes (previously diagnosed or not) and intermediate hyperglycemia for a diverse population of adults living in several Brazilian capital cities. Specifically, we highlighted a major aggregate burden of these conditions, particularly present in the elderly, in non-whites, in the obese and in those with less schooling. Since diabetes has been estimated to decrease the lifespan of middle-aged individuals by 6 years [27], these findings lend support to the current discourse of the need to address the challenges of diabetes in the 21^st^ century by allocating greater focus and resources to preventive actions known to be effective [1, 28].

This burden, which has increased in Brazil and elsewhere in parallel with the climbing rates of obesity and the aging of the population, is truly a public health disaster in slow motion. For low and middle income countries, including Brazil, in which the preventive focus remains largely directed toward infection diseases and problems of maternal and child health, few public health preventive actions are currently as important as that of developing and implementing a strategy to control the rise of obesity and diabetes. Finally, since diabetes is more frequent among the less privileged of society, such actions will additionally contribute to ameliorate health inequities.

In conclusion, with full accounting, the prevalence of diabetes and intermediate hyperglycemia is very high, indicating the necessity of public health and clinical preventive actions.

## References

[1] World Health Organization (2013). Global Action Plan for the Prevention and Control of Noncommunicable Diseases 2013-2020.

[2] Vos T, Flaxman AD, Naghavi M, Lozano R, Michaud C, Ezzati M, Shibuya K, Salomon JA, Abdalla S, Aboyans V, Abraham J, Ackerman I, Aggarwal R, Ahn SY, Ali MK, Alvarado M, Anderson HR, Anderson LM, Andrews KG, Atkinson C, Baddour LM, Bahalim AN, Barker-Collo S, Barrero LH, Bartels DH, Basáñez M-G, Baxter A, Bell ML, Benjamin EJ, Bennett D (2012). Years lived with disability (YLDs) for 1160 sequelae of 289 diseases and injuries 1990-2010: a systematic analysis for the Global Burden of Disease Study 2010. Lancet.

[3] International Diabetes Federation (2013). IDF Diabetes Atlas.

[4] Malerbi DA, Franco LJ (1992). Multicenter study of the prevalence of diabetes mellitus and impaired glucose tolerance in the urban Brazilian population aged 30–69 yr. Diabetes Care.

[5] Schmidt MI, Duncan BB, Azevedo e Silva G, Menezes AM, Monteiro CA, Barreto SM, Chor D, Menezes PR (2011). Chronic non-communicable diseases in Brazil: burden and current challenges. Lancet.

[6] Morris DH, Khunti K, Achana F, Srinivasan B, Gray LJ, Davies MJ, Webb D (2013). Progression rates from HbA1c 6.0–6.4% and other prediabetes definitions to type 2 diabetes: a meta-analysis. Diabetologia.

[7] Aquino EML, Barreto SM, Bensenor IM, Carvalho MS, Chor D, Duncan BB, Lotufo PA, Mill JG, Molina MDC, Mota ELA, Passos VMA, Schmidt MI, Szklo M (2012). Brazilian Longitudinal Study of Adult Health (ELSA-Brasil): objectives and design. Am J Epidemiol.

[8] Schmidt MI, Duncan BB, Mill JG, Lotufo PA, Chor D, Barreto SM, Aquino EM, Passos VMA, Matos SM, M d CB M, Carvalho MS, Bensenor IM (2014). Cohort profile: Longitudinal Study of Adult Health (ELSA-Brasil). Int J Epidemiol.

[9] Fedeli LG, Vidigal PG, Leite CM, Castilhos CD, Pimentel RA, Maniero VC, Mill JG, Lotufo PA, Pereira AC, Bensenor IM (2013). Logistics of collection and transportation of biological samples and the organization of the central laboratory in the ELSA-Brasil. Rev Saude Publica.

[10] World Health Organization (1999). Definition, Diagnosis and Classification Of Diabetes Mellitus and its Complications. Report of a WHO Consultation.

[11] American Diabetes Association (2014). Standards of medical care in diabetes: 2014. Diabetes Care.

[12] World Health Organization (2011). Use of Glycated Haemoglobin (HbA1c) in the Diagnosis of Diabetes Mellitus: Abbreviated Report of a WHO Consultation.

[13] World Health Organization (2006). Definition and Diagnosis of Diabetes Mellitus and Intermediate Hyperglycaemia: Report of a WHO/IDF Consulation.

[14] Gillett MJ (2009). International Expert Committee report on the role of the A1c assay in the diagnosis of diabetes: Diabetes Care 2009; 32(7): 1327-1334. Clin Biochem Rev Aust Assoc Clin Biochem.

[15] Schmidt MI, Duncan BB, Hoffmann JF, de Moura L, Malta DC, de Carvalho RMSV (2009). Prevalence of diabetes and hypertension based on self-reported morbidity survey, Brazil, 2006. Rev Saude Publica.

[16] Brasil. Ministério da Saúde (2014). Vigitel Brasil 2013: Vigilância de Fatores de Risco E Proteção Para Doenças Crônicas Por Inquérito Telefônico.

[17] Maruthur NM, Kao WHL, Clark JM, Brancati FL, Cheng C-Y, Pankow JS, Selvin E (2011). Does genetic ancestry explain higher values of glycated hemoglobin in African Americans?. Diabetes.

[18] Bullard KM, Saydah SH, Imperatore G, Cowie CC, Gregg EW, Geiss LS, Cheng YJ, Rolka DB, Williams DE, Caspersen CJ (2013). Secular changes in U.S. Prediabetes prevalence defined by hemoglobin A1c and fasting plasma glucose: National Health and Nutrition Examination Surveys, 1999-2010. Diabetes Care.

[19] Yang W, Lu J, Weng J, Jia W, Ji L, Xiao J, Shan Z, Liu J, Tian H, Ji Q, Zhu D, Ge J, Lin L, Chen L, Guo X, Zhao Z, Li Q, Zhou Z, Shan G, He J, China National Diabetes and Metabolic Disorders Study Group (2010). Prevalence of diabetes among men and women in China. N Engl J Med.

[20] Mc Donald PAJ, Montenegro GJA, Cruz GCE, Moreno de Rivera AL, Cumbrera OA (2013). Prevalence, sociodemographic distribution, treatment and control of diabetes mellitus in Panama. Diabetol Metab Syndr.

[21] Xu Y, Wang L, He J, Bi Y, Li M, Wang T, Wang L, Jiang Y, Dai M, Lu J, Xu M, Li Y, Hu N, Li J, Mi S, Chen C-S, Li G, Mu Y, Zhao J, Kong L, Chen J, Lai S, Wang W, Zhao W, Ning G, China Noncommunicable Disease Surveillance Group (2010). Prevalence and control of diabetes in Chinese adults. JAMA.

[22] Borch-Johnsen K, Colagiuri S, Balkau B, Glümer C, Carstensen B, Ramachandran A, Dong Y, Gao W (2004). Creating a pandemic of prediabetes: the proposed new diagnostic criteria for impaired fasting glycaemia. Diabetologia.

[23] Monteiro CA, Conde WL, Popkin BM (2007). Income-specific trends in obesity in Brazil: 1975-2003. Am J Public Health.

[24] Ferreira SR, Iunes M, Franco LJ, Iochida LC, Hirai A, Vivolo MA (1996). Disturbances of glucose and lipid metabolism in first and second generation Japanese-Brazilians. Japanese-Brazilian Diabetes Study Group. Diabetes Res Clin Pract.

[25] Selvin E, Crainiceanu CM, Brancati FL, Coresh J (2007). Short-term variability in measures of glycemia and implications for the classification of diabetes. Arch Intern Med.

[26] Instituto Brasileiro de Geografia e Estatística (2013). Atlas Do Censo Demográfico 2010.

[27] Seshasai SRK, Kaptoge S, Thompson A, Di Angelantonio E, Gao P, Sarwar N, Whincup PH, Mukamal KJ, Gillum RF, Holme I, Njølstad I, Fletcher A, Nilsson P, Lewington S, Collins R, Gudnason V, Thompson SG, Sattar N, Selvin E, Hu FB, Danesh J, Emerging Risk Factors Collaboration (2011). Diabetes mellitus, fasting glucose, and risk of cause-specific death. N Engl J Med.

[28] Zimmet PZ, Magliano DJ, Herman WH, Shaw JE (2014). Diabetes: a 21st century challenge. Lancet Diabetes Endocrinol.

